# Evaluation of endocan biomarker levels in peri-implant crevicular fluid of healthy and diseased peri-implant sites: a cross-sectional study

**DOI:** 10.1186/s12903-025-06633-8

**Published:** 2025-07-30

**Authors:** Ahmed I. Awad, Maha A. Abou Khadr, Sabah Abdelhady, Rania Abdelaziz

**Affiliations:** 1 Department of Oral Medicine, Periodontology, Oral Diagnosis and Oral Radiology, Division of periodontology, Faculty of Dentistry, Alexandria University, Champollion St, P.O. Box: 21523, Azarita, Alexandria Egypt; 2 Department of Medical Biochemistry, Faculty of Medicine, Alexandria University, alexandria, Egypt; 3 Department of Oral surgical sciences, division of periodontology, Beirut Arab University, Beirut, Lebanon

**Keywords:** Endocan, Peri-implantitis, Peri-implant mucositis, Biomarker, Peri-implant disease

## Abstract

**Objective:**

This investigation evaluates Endothelial Cell-Specific Molecule-1 (Endocan), an endothelial-derived protein, for its capacity to discriminate between healthy and pathological peri-implant tissues and its diagnostic potential in peri-implant disease.

**Materials and methods:**

This cross-sectional investigation analyzed 62 peri-implant sites from 62 individuals, including 31 healthy sites, chosen according to clinical and radiographic parameters based on 2017 consensus recommendations. Collection of peri-implant crevicular fluid (PICF) utilized paper point methodology, with Endocan quantification performed through enzyme-linked immunosorbent assay (ELISA) techniques. Statistical analysis included multivariable linear regression models to evaluate associations between disease status and Endocan levels, adjusting for demographic factors.

**Results:**

Endocan levels demonstrated a clear disease progression pattern. Healthy sites showed the lowest levels (643.66 ± 128.49 ng/L), while peri-implantitis sites exhibited significantly elevated concentrations (841.83 ± 62.72 ng/L, *p* < 0.001). Peri-implant mucositis sites (671.99 ± 133.00 ng/L) were not significantly different from healthy sites (*p* = 0.757). Modeling demonstrated that peri-implantitis exhibited significant correlation with increased Endocan concentrations (B = 187.74, *p* < 0.001), while peri-implant mucositis showed no significant association (*p* = 0.431). Additionally, younger age, female sex, and smoking were independently associated with higher Endocan concentrations.

**Conclusions:**

Endocan demonstrates specificity as a biomarker for peri-implantitis, showing capacity to differentiate advanced peri-implant pathology from healthy tissues and peri-implant mucositis. These results suggest Endocan’s potential utility for disease staging and monitoring in peri-implant care, providing a foundation for biomarker-based diagnostic approaches in implant dentistry.

## Introduction

Dental implants represent an established therapeutic modality for edentulous individuals, offering sustained functional and aesthetic advantages [1]. Nevertheless, biological complications, specifically peri-implant pathologies, constitute major obstacles in contemporary implant practice [2]. Peri-implant pathologies encompass peri-implant mucositis, defined by soft tissue inflammatory changes without osseous destruction, and peri-implantitis, characterized by progressive supporting bone destruction surrounding the implant [3, 4].

The pathogenesis of peri-implant diseases shares similarities with periodontitis, involving bacterial biofilm-induced inflammatory responses that lead to endothelial activation and leukocyte recruitment [5]. Traditional diagnostic methods, including clinical probing, bleeding on probing, and radiographic evaluation, have demonstrated limited sensitivity and specificity for early disease detection [6]. This limitation emphasizes the need for objective biomarkers to improve diagnosis and management of peri-implant conditions [7]. Endothelial Cell-Specific Molecule-1 (Endocan), also known as ESM-1, is a soluble proteoglycan produced by endothelial cells that plays crucial roles in inflammation, angiogenesis, and leukocyte migration [8]. Endocan participates in various biological processes including cell proliferation, neovascularization, and the expression of cell adhesion molecules such as VCAM-1 and ICAM-1 [9]. Elevated Endocan levels have beenassociated with various inflammatory conditions, including cardiovascular diseases, sepsis, and cancer [10, 11]. Recent studies have demonstrated increased Endocan levels in periodontal disease, suggesting its potential role as an inflammatory biomarker in oral tissues [12, 13]. Given the pathological similarities between periodontitis and peri-implant diseases, Endocan may serve as a valuable biomarker for detecting and monitoring peri-implant inflammation.

However, minimal research has examined Endocan concentrations in peri-implant tissues, and its potential for distinguishing between different stages of peri-implant disease remains unexplored.

### Study hypothesis

The null hypothesis of this study assumes that there is no significant variation in Endocan concentrations between healthy and pathological peri-implant locations.

### Study aims

#### Primary aim

To assess and compare Endocan values in peri-implant crevicular fluid (PICF) between healthy and diseased peri-implant locations.

### Secondary aims

1. To assess whether Endocan levels differ between peri-implant mucositis and peri-implantitis.
2. To investigate potential correlations between Endocan levels and demographic factors (age, smoking status) in diseased peri-implant locations.
3. To evaluate the potential of Endocan as a diagnostic biomarker for peri-implant disease.

## Methodology

### Patient selection

This investigation was conducted involving individuals with previously placed dental implants recruited from the outpatient department at the Faculty of Dentistry, Alexandria University during October 2023 through May 2024. This clinical investigation analyzed sixty-two selected locations classified into two categories according to clinical and radiographic assessments. The investigation’s objectives and methodology were explained, and informed agreement was secured from participating individuals before any procedures [14]. Participants who presented to the periodontology outpatient department for implant assessment or monitoring were enrolled in the investigation.

The 1975 Declaration of Helsinki guidelines were adhered to. Ethical clearance for the investigation was secured.

A cross-sectional investigation adhering to STROBE recommendations.

### Diagnostic criteria (Following 2017 consensus Reports) [15]

#### Peri-implant health

Peri-implant Health:

- No clinical inflammatory indicators.
- Absence of bleeding upon probing (BOP).
- No purulent discharge.
- Bone loss not surpassing initial remodeling (≤ 2 mm from baseline radiographs).

#### Peri-implant mucositis

- Clinical inflammatory signs (erythema, edema).
  - (BOP) and/or purulent discharge.
  - No osseous loss exceeding initial remodeling compared to baseline radiographs.
- Probing depth may be increased compared to baseline.

#### Peri-implantitis

- Clinical inflammatory indicators.
  - Bleeding upon probing and/or purulent discharge.
  - Radiographic bone loss ≥ 3 mm from baseline radiographs, OR.
- In absence of baseline radiographs: bone loss ≥ 3 mm coupled with probing depth ≥ 6 mm.

### Inclusion criteria

- Age ≥ 18 years.
  - Presence of minimum one endosseous dental implant in oral cavity.
  - Minimum one year following prosthetic installation.
  - Clinical and radiographic evidence of healthy or pathological implant conditions.
  - Systemically healthy according to American Society of Anesthesiologists classification I (ASA I).
- Ability to perform adequate oral hygiene procedures.
- Willingness to participate and provide informed consent.

### Exclusion criteria

- Pregnancy or breastfeeding.
- Antibiotic or anti-inflammatory medication use within three months before enrollment.
  - Periodontal or peri-implant therapy within preceding six months.
  - Systemic disorders affecting periodontal health (uncontrolled diabetes mellitus, rheumatoid arthritis).
- Incapacity for standard oral hygiene maintenance.

### Peri-implant crevicular fluid (PICF) collection

PICF samples were obtained prior to clinical assessments to prevent contamination.

The collection protocol included:

- Isolation of the dental implant site with cotton rolls.
- Gentle air drying of the area.
- Placement of sterile paper points within the peri-implant sulcus until mild resistance was encountered.
- Paper points maintained in position for 30 s.
- Blood-contaminated samples were excluded.
- Paper points were promptly placed in coded Eppendorf tubes containing phosphate buffer saline solution.
- Samples were stored at -80 °C pending analysis.

#### Sample size

A specimen count of 62 peri-implant tissue samples, maintaining a 1:1 proportion of healthy to pathological groups (minimum 31 pathological peri-implant tissues), was established as adequate for diagnostic test accuracy investigation. Assumptions encompassed a significance threshold of 5% (α error = 0.05), statistical power (1-β) of 80%, and discrimination threshold (area under ROC) of 70% [16].

#### Grouping

Group I encompassed 31 sites with healthy peri-implant tissue (control group), whereas.

Group II included 31 sites with pathological peri-implant tissues.

### Clinical measurements

**Fig. 1:**
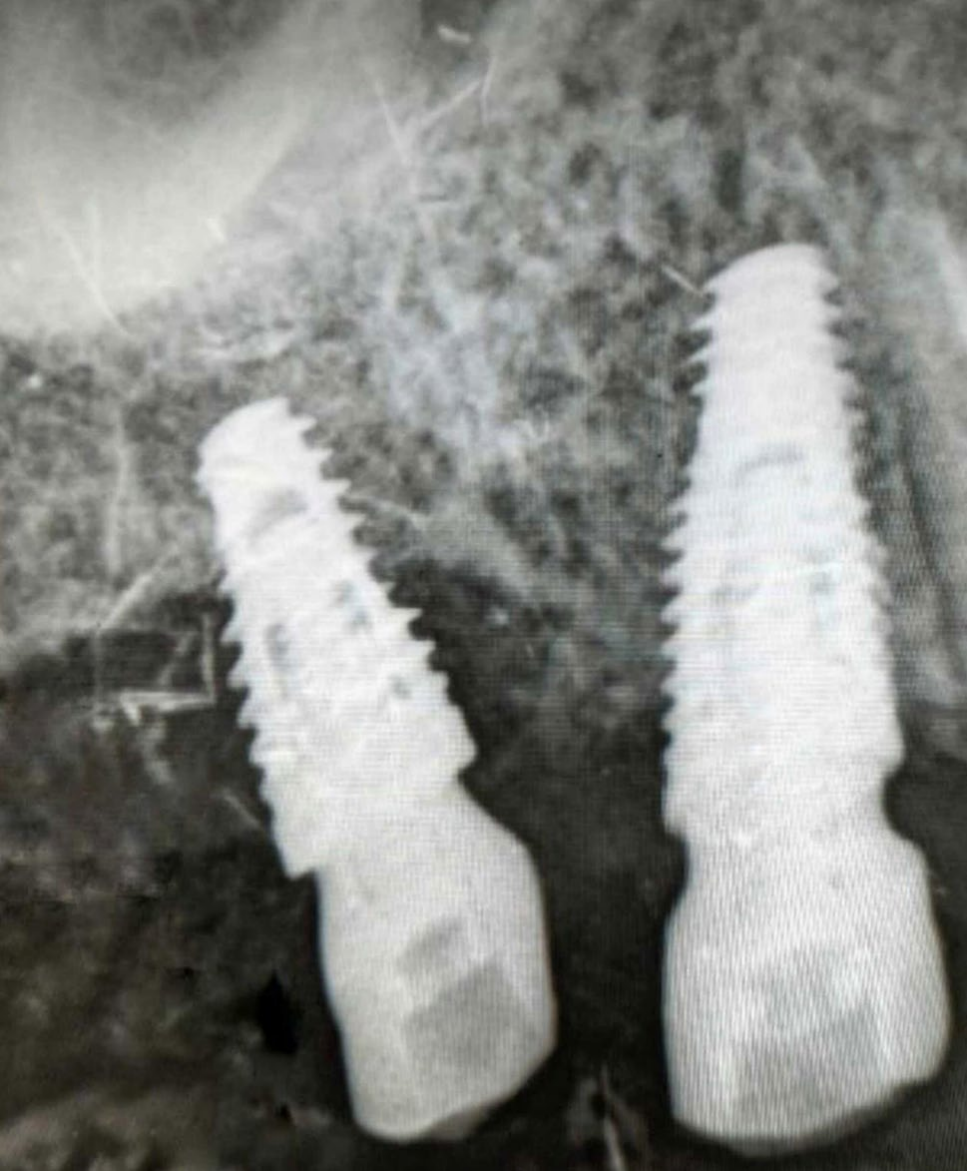
Radiographic assessment of peri-apical radiographic bone status

Peri-implant probing depth assessments were performed at six locations (mesiobuccal, midbuccal, distobuccal, mesiolingual, mid-lingual, and distolingual) surrounding healthy and pathological implants utilizing a plastic periodontal probe [17]. Suppuration was assessed as purulent exudate from the gingival sulcus upon probing or digital pressure along the gingiva of the corresponding dental implant [15]. Radiographic bone level evaluation was conducted utilizing standardized periapical radiographs with parallel methodology. Marginal bone loss was quantified from the implant shoulder (implantabutment junction) to the initial point of bone-to-implant contact [18].(Fig. 1).

Endocan concentrations were established through collection of (PICF) utilizing paper points for evaluation. (PICF) specimens were obtained prior to clinical assessments. During specimen collection, the implant was isolated using dental cotton rolls, gently desiccated using air spray, and paper strips were introduced into the pocket until resistance was encountered, maintained for 30 s and continued until mild resistance was identified.

**Fig. 2:**
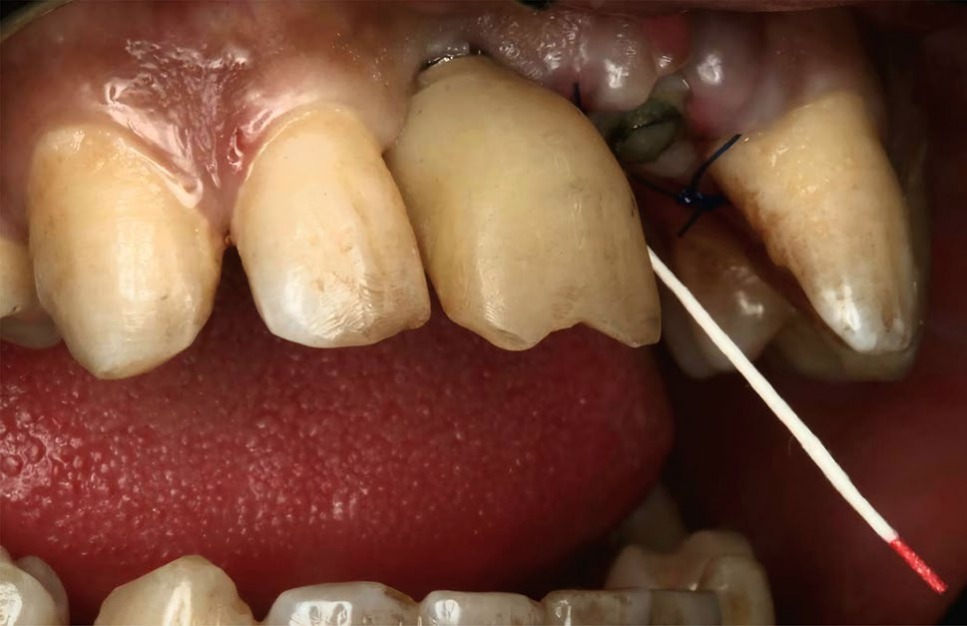
(PICF) samples collection

**Fig. 3:**
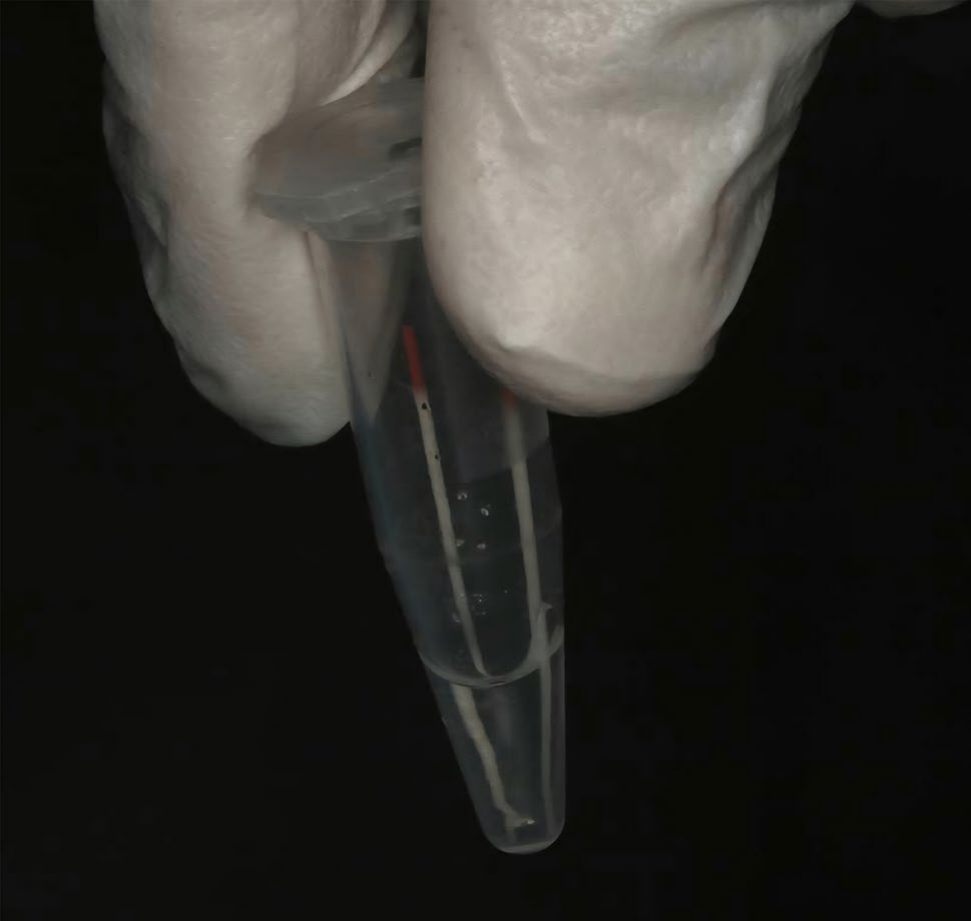
The paper points were storage in Eppendorfs with phosphate buffer saline solution

Blood-contaminated strips were excluded [19].(Fig. 2)The paper points were stored in Eppendorfs with phosphate buffer saline solution that had already been encoded, maintained at -80 degrees until analysis, and measurements were recorded [20] (Fig. 3).

### PICF ELISA method for endocan

Endocan levels in peri-implant crevicular fluid (PICF) samples were analyzed using the Human ECSM1/ENDOCAN ELISA kit (Catalogue No. 201-12-1978). All assay procedures were carried out in accordance with the manufacturer’s specifications.

### Measurement reliability

Intra-examiner reliability for radiographic bone loss measurements was assessed using the intraclass correlation coefficient (ICC). The reliability analysis demonstrated excellent measurement consistency with an ICC of 0.977 (95% CI: 0.942–0.991, *p* < 0.001).

### Statistical analysis

#### Statistical analysis of the data

Normality of quantitative variables (age and Endocan levels) was evaluated using the Shapiro–Wilk test and Q–Q plots, verifying normal distribution. subsequently, quantitative data were summarized using mean and standard deviation (SD), while qualitative variables were presented as frequencies and percentages. Group comparisons were performed using the independent t-test and one-way ANOVA, followed by Scheffé’s post hoc test with Bonferroni correction. Pearson’s chi-square test was used for categorical comparisons. Two multivariable linear regression models were developed to evaluate the association between disease status and Endocan levels, adjusting for potential confounders including age, sex, smoking status, and diabetic condition. Regression coefficients (B), 95% confidence intervals (CI), and model summaries were reported. All analyses were two-tailed, and a p-value < 0.05 was deemed statistically significant. Intra examiner reliability was assessed using Intraclass Correlation coefficient (ICC). Data analysis was preformed using IBM SPSS Statistics for Windows, version 23.0 (Armonk, NY: IBM Corp).

## Results

### Study population and demographics

A total of 62 peri-implant sites from 62 participants were included in this study, comprising 31 healthy peri-implant sites and 31 diseased sites. The diseased sites were further classified into 18 peri-implantitis sites and 13 peri-implant mucositis sites based on radiographic bone loss and clinical parameters.

The demographic characteristics of the study participants are displayed in Table 1. No statistically significant differences were detected between healthy and diseased groups No statistically significant differences were observed between healthy and diseased groups regarding age (39.42 ± 9.79 vs. 37.90 ± 8.92 years, *p* = 0.526), gender distribution (*p* = 0.562), smoking status (*p* = 0.799), or diabetes prevalence (*p* = 0.113). When comparing the three groups (healthy, peri-implantitis, and peri-implant mucositis), the demographic distributions remained similar with no significant differences (*p* > 0.05 for all comparisons).

**Table 1:** Demographic and characteristics of the study participants

		Healthy (*n* = 31)	Diseased			*P* value^1^ (*P* value^2^)
			Peri-implantitis (*n* = 18)	peri-implant mucositis (*n* = 13)	Overall (*n* = 31)	
Age in years	Mean ± SD	39.42 ± 9.79	35.85 ± 6.66	39.39 ± 10.18	37.90 ± 8.9	0.526 (0.480)
Gender: n (%)	Males	24 (77.4%)	11 (84.6%)	11 (61.1%)	22 (71%)	0.562 (0.284)
	Females	7 (22.6%)	2 (15.4%)	7 (38.9%)	9 (29%)	
Smoking: n (%)	Yes	17 (54.8%)	5 (38.5%)	11 (61.1%)	16 (51.6%)	0.799 (0.445)
	No	14 (45.2%)	8 (61.5%)	7 (38.9%)	15 (48.4%)	
Diabetes: n (%)	Yes	0 (0%)	1 (7.7%)	3 (16.7%)	4 (12.9%)	0.113 (0.071)
	No	31 (100%)	12 (92.3%)	15 (83.3%)	27 (87.1%)	

*P* value^1^ is obtained based on comparison between healthy and all diseased, *P* value^2^ is obtained based on comparison between healthy and the two subgroups of diseased

### Clinical parameters

#### Bone loss measurements

Healthy sites demonstrated minimal bone loss (1.53 ± 0.46 mm), while diseased sites overall showed significantly greater bone loss (3.10 ± 1.24 mm, *p* < 0.001). When examining disease subtypes, peri-implantitis sites exhibited the highest bone loss (4.00 ± 0.77 mm), which was significantly different from both healthy sites (*p* < 0.001) and peri-implant mucositis sites (*p* < 0.001). Peri-implant mucositis sites showed bone loss values (1.85 ± 0.32 mm) that were not significantly different from healthy sites (*p* = 0.230) (Table 2).

**Table 2:** Comparison of bone loss (mm) and probing depth (mm) between healthy and disease peri-implant sites

		Healthy (*n* = 31)	Diseased			*P* value^1^ (*P* value^2^)
			Peri-implantitis (*n* = 18)	peri-implant mucositis (*n* = 13)	Overall (*n* = 31)	
Bone loss	Mean ± SD	1.53 ± 0.46	4.00 ± 0.77	1.85 ± 0.32	3.10 ± 1.24	< 0.001* (< 0.001*)
Pairwise comparison	Healthy vs. Peri-implantitis < 0.001* Healthy vs. peri-implant mucositiss = 0.230 Peri-implantitis vs. peri-implant mucositis < 0.001*					
Probing depth	Mean ± SD	3.32 ± 0.79	6.94 ± 0.87	4.77 ± 0.44	6.03 ± 1.30	< 0.001* (< 0.001*)
Pairwise comparison	Healthy vs. Peri-implantitis < 0.001* Healthy vs. peri-implant mucositis < 0.001* Peri-implantitis vs. peri-implant mucositis < 0.001*					

*Statistically significant difference at *p* value < 0.05, *P* value^1^ is obtained based on comparison between healthy and all diseased, *P* value^2^ is obtained based on comparison between healthy and the two subgroups of diseased

#### Probing depth measurements

Probing depth measurements showed healthy sites with depths of 3.32 ± 0.79 mm, peri-implant mucositis sites with 4.77 ± 0.44 mm, and peri-implantitis sites with 6.94 ± 0.87 mm. All pairwise comparisons between the three groups were statistically significant (*p* < 0.001 for all comparisons) (Table 2).

#### Endocan levels

Endocan concentrations in peri-implant crevicular fluid are presented in Table 3. Healthy peri-implant sites demonstrated Endocan levels of 643.66 ± 128.49 ng/L, while diseased sites overall showed levels of 770.61 ± 128.70 ng/L (*p* < 0.001).

**Table 3:** Comparison of endocan levels between the healthy and diseases patients

	Healthy (*n* = 31)	Diseased patients		
		Peri-implantitis (*n* = 13)	peri-implant mucositis (*n* = 18)	Overall (*n* = 31)
Mean ± SD	643.66 ± 128.49	841.83 ± 62.72	671.99 ± 133.00	770.61 ± 128.70
*P* value^1^ (*P* value^2^)	< 0.001* (0.001*)			
Pairwise comparisons	Healthy vs. Peri-implantitis < 0.001* Healthy vs. peri-implant mucositis = 0.757 Peri-implantitis vsperi-implant mucositis = 0.001*			

*Statistically significant difference at *p* value < 0.05, *P* value^1^ is obtained based on comparison between healthy and all diseased, *P* value^2^ is obtained based on comparison between healthy and the two subgroups of diseased

**Fig. 4:**
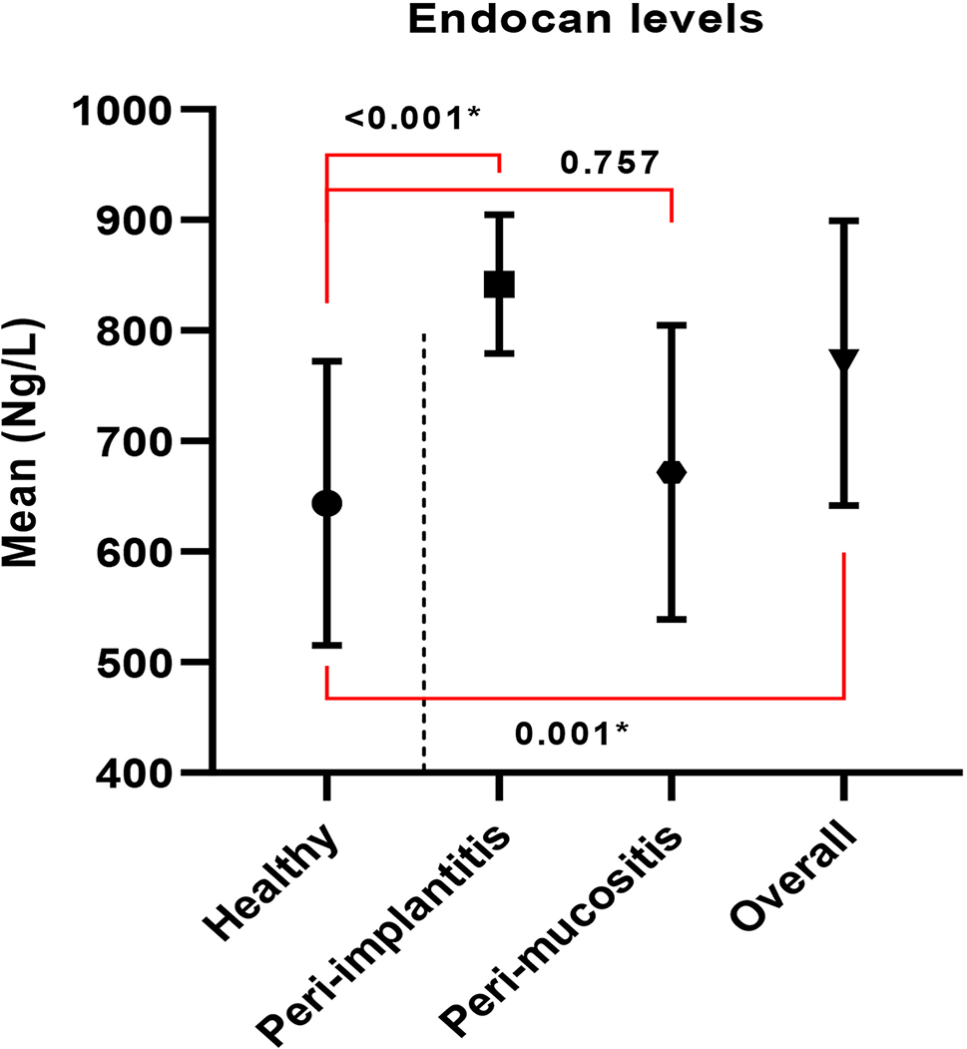
Comparison between the three studied groups according to Endocan

When examining disease subtypes, peri-implantitis sites exhibited Endocan concentrations of 841.83 ± 62.72 ng/L, which were significantly different from both healthy sites (*p* < 0.001) and peri-implant mucositis sites (*p* = 0.001). Peri-implant mucositis sites showed Endocan levels of 671.99 ± 133.00 ng/L, which were not significantly different from healthy sites (*p* = 0.757); (Fig. 4).

### Multivariable regression analysis

Two multivariable linear regression models were constructed to evaluate the association between various factors and Endocan levels (Table 4). Both models demonstrated excellent fit with the data, with Model 1 (χ² = 23.68, df = 5, *p* < 0.001) and Model 2 (χ² = 37.53, df = 6, *p* < 0.001) showing high statistical significance.

**Table 4:** Multivariable linear regression analyses assessing the effect of demographic and participant characteristics on endocan levels

Parameters	Model 1			Model 2		
	Coefficient (B)	95% CI	*p* value	Coefficient (B)	95% CI	*p* value
Age	-3.55	-7.04, -0.06	0.046*	-3.55	-6.69, -0.44	0.025*
Females vs. males	84.25	11.11, 157.40	0.042*	73.34	7.70, 138.99	0.029*
Smokers vs. Non-smokers	85.63	17.64, 153.62	0.014*	69.66	8.34, 130.99	0.026*
Diabetic vs. Non-diabetic	68.65	-56.38, 193.67	0.282	-10.90	-129.52, 107.72	0.867
Diseased vs. Healthy	110.03	49.50, 170.56	< 0.001*	-	-	-
Peri-implantitis vs. Healthy	-	-	-	187.74	121.22, 254.26	< 0.001*
peri-implant mucositis vs. Healthy	-	-	-	27.32	-40.68, 95.32	0.431

*Statistically significant difference at *p* value < 0.05, Model 1 includes general disease status (Diseased vs. Healthy); Model 2 includes disease subtypes (Peri-implantitis and peri-implant mucositis vs. Healthy), Model 1 summary: *X*^2^ = 23.68, df = 5, *p* value < 0.001*, Model 2 summary: *X*^2^ = 37.53, df = 6, *p* value < 0.001*

In Model 1, younger age (B = − 3.55, 95% CI: − 7.04 to − 0.06, *p* = 0.046), female sex (B = 84.25, 95% CI: 11.11 to 157.40, *p* = 0.042), and smoking (B = 85.63, 95% CI: 17.64 to 153.62, *p* = 0.014) were significantly associated with higher Endocan levels. Individuals with peri-implant disease showed markedly elevated Endocan levels compared to healthy participants (B = 110.03, 95% CI: 49.50 to 170.56, *p* < 0.001). In Model 2, these associations remained significant for age (B = − 3.55, 95% CI: − 6.69 to − 0.44, *p* = 0.025), female sex (B = 73.34, 95% CI: 7.70 to 138.99, *p* = 0.029), and smoking (B = 69.66, 95% CI: 8.34 to 130.99, *p* = 0.026). Peri-implantitis was strongly associated with higher Endocan levels (B = 187.74, 95% CI: 121.22 to 254.26, *p* < 0.001), whereas peri-implant mucositis was not statistically significant (*p* = 0.431). Diabetes was not a significant predictor in either mod

In Model 1, younger age (B = − 3.55, 95% CI: − 7.04 to − 0.06, *p* = 0.046), female sex (B = 84.25, 95% CI: 11.11 to 157.40, *p* = 0.042), and smoking (B = 85.63, 95% CI: 17.64 to 153.62, *p* = 0.014) were significantly associated with higher Endocan levels. Individuals with peri-implant disease showed elevated Endocan levels compared to healthy participants (B = 110.03, 95% CI: 49.50 to 170.56, *p* < 0.001).

In Model 2, these associations remained significant for age (B = − 3.55, 95% CI: − 6.69 to − 0.44, *p* = 0.025), female sex (B = 73.34, 95% CI: 7.70 to 138.99, *p* = 0.029), and smoking (B = 69.66, 95% CI: 8.34 to 130.99, *p* = 0.026). Peri-implantitis was associated with higher Endocan levels (B = 187.74, 95% CI: 121.22 to 254.26, *p* < 0.001), whereas peri-implant mucositis was not statistically significant (*p* = 0.431). Diabetes was not a significant predictor in either model.

## Discussion

The search for reliable biomarkers in peri-implant disease has long been hampered by the challenge of distinguishing between different disease stages. This study provides the first evidence that Endocan levels in peri-implant crevicular fluid can specifically distinguish peri-implantitis from both healthy sites and peri-implant mucositis, addressing a critical need in implant diagnostics. These results add to growing interest in site-specific biomarkers capable of reflecting distinct disease stages.

Our investigation reveals a compelling pattern of biomarker specificity that sets Endocan apart from conventional inflammatory markers. We demonstrate significantly elevated Endocan concentrations in peri-implantitis sites (841.83 ± 62.72 ng/L) compared to healthy sites (643.66 ± 128.49 ng/L, *p* < 0.001), while peri-implant mucositis sites showed no significant elevation (*p* = 0.757). This selective elevation was further validated through multivariable regression analysis, which confirmed that peri-implantitis was strongly associated with elevated Endocan (B = 187.74, *p* < 0.001) while peri-implant mucositis was not (*p* = 0.431). This pattern indicates that Endocan reflects endothelial dysfunction characteristic of established tissue destruction rather than early inflammatory changes, suggesting its potential as a marker of disease severity rather than mere presence of inflammation.

Our findings indicate that Endocan levels may show a different pattern compared to conventional PICF biomarkers, suggesting a potential association with specific disease stages. Our Endocan findings contrast markedly with traditional inflammatory mediators, where Unlike IL-1β and TNF-α which typically elevate in both mucositis and peri-implantitis (Dursun and Tözüm, 2016) [10], Endocan demonstrated specificity for peri-implantitis only. This distinction becomes even more apparent when comparing our results to recent comprehensive studies. While Li et al. (2022) reported substantial increases in IL-1β, IL-6, and IL-17 A in peri-implantitis [21], our observed Endocan elevation was more modest, reflecting its specific role as an endothelial dysfunction marker rather than a general inflammatory mediator.

The pattern of biomarker specificity extends beyond individual cytokines to broader inflammatory cascades. Similarly, Faot et al. (2015) demonstrated elevated inflammatory biomarkers including MMP-8 in peri-implantitis, and like RANKL/OPG ratios, these markers typically rise in both disease stages, whereas Endocan showed specificity for advanced disease only [22]. This selective elevation suggests that Endocan may serve as a more precise diagnostic tool for disease staging than currently available biomarkers.

The relevance of our findings extends beyond peri-implant disease to the broader context of oral inflammatory conditions. These findings align with periodontal disease research where Endocan elevation in periodontitis according to Balta et al., 2014 [23] and its role as a diagnostic/prognostic marker according to Kumar et al., 2019; Gopakumar et al., 2023 [24, 25] support its relevance as a vascular inflammatory biomarker in oral diseases. However, our results also reveal important distinctions in biomarker behavior across different oral tissues and disease contexts. Interestingly, while Ayad et al. (2020) demonstrated Endocan correlation with bleeding on probing in diabetic periodontitis patients [26], our study found no significant diabetes association (*p* = 0.867), suggesting tissue-specific or disease-stage-dependent responses.

Conversely, our observed smoking association (B = 69.66, *p* = 0.026) aligns with established effects on endothelial function, reinforcing the biological plausibility of Endocan as an endothelial dysfunction marker. The methodological strength of our approach becomes apparent when considering the limitations of previous research. Importantly, our three-group analysis addresses a critical gap, as many earlier PICF studies did not differentiate mucositis from peri-implantitis, limiting clinical interpretation. Our study helps bridge this gap, suggesting that Endocan may enhance diagnostic precision and support clinical staging, treatment planning, and disease monitoring.

### Clinical findings summary

The clinical implications of our findings emerge from the clear disease progression pattern observed across multiple parameters. The results demonstrate a clear disease progression pattern in both clinical parameters and biomarker levels, with each disease stage showing distinct characteristics. Peri-implantitis sites consistently showed the most severe clinical presentation with the highest bone loss, deepest probing depths, and most elevated Endocan levels. In contrast, Peri-implant mucositis sites presented with intermediate clinical parameters but Endocan levels that were not significantly different from healthy sites, suggesting that Endocan elevation occurs specifically with the transition to destructive disease.

The robustness of these associations was confirmed through comprehensive statistical modeling. The multivariable regression analysis revealed that peri-implantitis, but not peri-implant mucositis, was independently associated with elevated Endocan levels after adjusting for demographic and clinical factors. This independence from confounding variables strengthens the clinical utility of Endocan as a diagnostic marker. Additionally, younger age, female sex, and smoking status emerged as significant predictors of higher Endocan concentrations in peri-implant crevicular fluid, providing insights into patient-specific factors that may influence biomarker interpretation in clinical practice.

### Rationale and evaluation of PICF sampling

Peri‑implant crevicular fluid (PICF) offers key diagnostic advantages. It is the most site-specific biological fluid available, collected directly from the implant sulcus and accurately reflecting local tissue conditions [19]. Systematic reviews have demonstrated that PICF contains inflammatory cytokines such as IL-1β and TNF-α that can assist in the diagnosis of peri-implant diseases [22, 27]. Compared to alternatives, PICF offers a superior balance between diagnostic accuracy and practicality. Saliva is easier to collect but lacks site specificity and is subject to dilution. Serum reflects systemic conditions, not localized inflammation. Tissue biopsies provide detailed local information but are invasive and not suitable for routine monitoring [19].

Despite limitations such as small sample volume and operator sensitivity, standardized collection techniques such as filter paper strips enhance reproducibility and clinical feasibility.

Thus, PICF represents a minimally invasive, real-time diagnostic medium particularly well suited to biomarker-based diagnostics like Endocan, especially for staging and monitoring peri-implant disease.

### Study limitations

This study’s cross-sectional design restricts causal inference; we cannot determine whether elevated Endocan levels precede or follow disease onset. Longitudinal studies are needed to explore biomarker dynamics over time. While the sample size allowed meaningful comparisons between healthy and diseased sites, it limited subgroup analyses. Future multicenter studies with larger cohorts are necessary to enhance generalizability and control for confounding variables.

Our smoking classification was based on current use only, with former smokers considered non-smokers, and no data on intensity, duration, or pack-years, restricting interpretation of dose-dependent effects. Heavy smokers were excluded to reduce confounding, but this limits applicability to this high-risk population. Future research should adopt standardized smoking classifications to capture more nuanced associations.

The absence of baseline radiographs limited our ability to assess bone remodeling patterns from implant placement. While diagnostic criteria followed 2017 consensus guidelines [15], incorporating baseline imaging would strengthen future analyses.

Clinical measurements included probing depth and radiographic bone loss but excluded bleeding on probing, attachment loss, and suppuration. While our selected parameters are core diagnostic criteria, a broader clinical assessment could enhance diagnostic accuracy [15].

### Future directions

Further validation through larger, multicenter, and longitudinal studies is needed to confirm Endocan’s utility in disease prediction, monitoring, and treatment response. Future research should investigate Endocan levels in heavy smokers and individuals with systemic conditions to explore influencing variables. The development of chairside Endocan assays would support real-time clinical decisions and advance personalized management in peri-implant care.

## Conclusion

- This study provides the first evidence that PICF Endocan is a specific biomarker for peri-implantitis, with promising applications in disease staging and monitoring. Its site-specific, non-invasive nature supports its potential integration into routine implant diagnostics, pending validation in larger prospective trials.
- Endocan concentrations were significantly increased in peri-implantitis relative to healthy locations, demonstrating strong association with inflammatory activity.
- Endocan demonstrates potential as a diagnostic biomarker for peri-implant pathology.
- Potentially supplementing or improving upon clinical and radiographic methods. The findings support a vascular-inflammatory component in peri-implant pathology, highlighting endothelial dysfunction.
- While Endocan distinguishes between healthy and peri-implantitis, its capacity to differentiate mucositis from implantitis requires further investigation. Systemic factors such as diabetes and smoking should be explored in larger, longitudinal studies.

Continued research is required to assess Endocan’s capacity as an early pathological indicator and a mechanism for monitoring therapeutic response in peri-implant management.

### Future research directions

Continued investigation is required to assess Endocan’s capacity as an early pathological indicator and a mechanism for monitoring therapeutic response in peri-implant management.

## Data Availability

The datasets created and analyzed during this study can be obtained from the corresponding author upon reasonable request.

## Abbreviations

ESM-1: Endothelial cell molecule
PMNLs: Polymorphonuclear leukocytes
PICF: Peri implant crevicular fluid
GICF: Gingival crevicular fluid
BOP: Bleeding on probing
CAM: Cell adhesion molecule

## References

[1] Chappuis V, Buser R, Brägger U, Bornstein MM, Salvi GE, Buser D. Long-term outcomes of dental implants with a titanium plasma-sprayed surface: a 20-year prospective case series study in partially edentulous patients. Clin Implant Dent Relat Res. 2013;15:780–90. 10.1111/cid.12056. 23506385 10.1111/cid.12056

[2] Lang NP, Berglundh T. Periimplant diseases: where are we now?>--Consensus of the seventh European workshop on periodontology. J Clin Periodontol. 2011;38(Suppl 11):178–81. 10.1111/j.1600-051X.2010.01674.x. 10.1111/j.1600-051X.2010.01674.x 21323713

[3] Lindhe J, Meyle J. Peri-implant diseases: consensus report of the sixth European workshop on periodontology. J Clin Periodontol. 2008;35:282–5. 10.1111/j.1600-051X.2008.01283.x. 18724855 10.1111/j.1600-051X.2008.01283.x

[4] Greenstein G, Eskow R. High prevalence rates of Peri-implant mucositis and Peri-implantitis post dental implantations dictate need for continuous Peri-implant maintenance. Compend Contin Educ Dent. 2022;43:206–13. quiz 14. 35380854

[5] Muller WA. Leukocyte-endothelial-cell interactions in leukocyte transmigration and the inflammatory response. Trends Immunol. 2003;24:327–34. 10.1016/s1471-4906(03)00117-0. 12810109 10.1016/s1471-4906(03)00117-0

[6] Del Fabbro M, Francetti L, Pizzoni L, Weinstein RL. [Congenital neutrophil defects and periodontal diseases]. Minerva Stomatol. 2000;49:293–311. 11189956

[7] Deas DE, Mackey SA, McDonnell HT. Systemic disease and periodontitis: manifestations of neutrophil dysfunction. Periodontol 2000. 2003;32:82–104. 10.1046/j.0906-6713.2003.03207.x. 12756035 10.1046/j.0906-6713.2003.03207.x

[8] Heitz-Mayfield LJ. Peri-implant diseases: diagnosis and risk indicators. J Clin Periodontol. 2008;35:292–304. 10.1111/j.1600-051X.2008.01275.x. 18724857 10.1111/j.1600-051X.2008.01275.x

[9] Ramseier CA, Kinney JS, Herr AE, Braun T, Sugai JV, Shelburne CA, et al. Identification of pathogen and host-response markers correlated with periodontal disease. J Periodontol. 2009;80:436–46. 10.1902/jop.2009.080480. 19254128 10.1902/jop.2009.080480 PMC5695217

[10] Dursun E, Tözüm TF. Peri-Implant crevicular fluid analysis, enzymes and biomarkers: a systemetic review. J Oral Maxillofac Res. 2016;7:e9. 10.5037/jomr.2016.7309. 27833734 10.5037/jomr.2016.7309 PMC5100649

[11] Hirano T, Akira S, Taga T, Kishimoto T. Biological and clinical aspects of Interleukin 6. Immunol Today. 1990;11:443–9. 10.1016/0167-5699(90)90173-7. 2127356 10.1016/0167-5699(90)90173-7

[12] Feghali CA, Wright TM. Cytokines in acute and chronic inflammation. Front Biosci. 1997;2:d12–26. 10.2741/a171. 9159205 10.2741/a171

[13] Schwarz F, Derks J, Monje A, Wang HL. Peri-implantitis. J Periodontol. 2018;89(Suppl 1):S267–90. 10.1002/jper.16-0350. 29926957 10.1002/JPER.16-0350

[14] Albrektsson T, Zarb G, Worthington P, Eriksson AR. The long-term efficacy of currently used dental implants: a review and proposed criteria of success. Int J Oral Maxillofac Implants. 1986;1:11–25. 3527955

[15] Renvert S, Persson GR, Pirih FQ, Camargo PM. Peri-implant health, peri-implant mucositis, and peri-implantitis: case definitions and diagnostic considerations. J Periodontol. 2018;89(Suppl 1):S304–12. 10.1002/jper.17-0588. 29926953 10.1002/JPER.17-0588

[16] Pjetursson BE, Asgeirsson AG, Zwahlen M, Sailer I. Improvements in implant dentistry over the last decade: comparison of survival and complication rates in older and newer publications. Int J Oral Maxillofac Implants. 2014;29. Suppl:308– 24 10.11607/jomi.2014suppl.g5.2. 10.11607/jomi.2014suppl.g5.2 24660206

[17] Brägger U, Bürgin WB, Hämmerle CH, Lang NP. Associations between clinical parameters assessed around implants and teeth. Clin Oral Implants Res. 1997;8:412–21. 10.1034/j.1600-0501.1997.080508.x. 9612146 10.1034/j.1600-0501.1997.080508.x

[18] Abreu MH, Bianchini MA, Magini RS, Rösing CK. Clinical and radiographic evaluation of periodontal and peri-implant conditions in patients with implant-supported prosthesis. Acta Odontol Latinoam. 2007;20:87–95. 18590257

[19] Griffiths GS. Formation, collection and significance of gingival crevice fluid. Periodontol 2000. 2003;31:32–42. 10.1034/j.1600-0757.2003.03103.x. 12656994 10.1034/j.1600-0757.2003.03103.x

[20] Pradeep AR, Raghavendra NM, Prasad MV, Kathariya R, Patel SP, Sharma A. Gingival crevicular fluid and serum visfatin concentration: their relationship in periodontal health and disease. J Periodontol. 2011;82:1314–9. 10.1902/jop.2011.100690. 21309715 10.1902/jop.2011.100690

[21] Liu YC, Lerner UH, Teng YT. Cytokine responses against periodontal infection: protective and destructive roles. Periodontol 2000. 2010;52:163–206. 10.1111/j.1600-0757.2009.00321.x. 20017801 10.1111/j.1600-0757.2009.00321.x

[22] Faot F, Nascimento GG, Bielemann AM, Campão TD, Leite FR, Quirynen M. Can peri-implant crevicular fluid assist in the diagnosis of peri-implantitis? A systematic review and meta-analysis. J Periodontol. 2015;86:631–45. 10.1902/jop.2015.140603. 25675962 10.1902/jop.2015.140603

[23] Balta S, Mikhailidis DP, Demirkol S, Ozturk C, Celik T, Iyisoy A, Endocan. A novel inflammatory indicator in cardiovascular disease? Atherosclerosis. 2015;243:339–43. 10.1016/j.atherosclerosis.2015.09.030. 26448266 10.1016/j.atherosclerosis.2015.09.030

[24] Kumar G, Ponnaiyan D, Parthasarathy H, Tadepalli A, Veeramani S. Evaluation of endocan and tumor necrosis Factor-α as inflammatory biomarkers in type 2 diabetes and periodontal disease. Genet Test Mol Biomarkers. 2020;24:431–5. 10.1089/gtmb.2020.0037. 32513032 10.1089/gtmb.2020.0037

[25] Gopakumar D, Arunima PR, Jacob Raja SA, Fairlin P. Gingival gene expression of endocan as biomarker of periodontitis and diabetes Mellitus– An in vivo comparative analysis. J Complement Herb Res. 2023;12:1–9.

[26] Ayad ES, Khadr A, Abd El Aziz M, Ayad MW, El-Kimary GI, Endocan. A novel biomarker in gingival crevicular fluid in periodontitis patients with or without type 2 diabetes mellitus [a cross-sectional study]. Alex Dent J. 2024;49:7–13. 10.21608/adjalexu.2023.201324.1360.

[27] Gürlek Ö, Gümüş P, Nile CJ, Lappin DF, Buduneli N. Biomarkers and Bacteria around implants and natural teeth in the same individuals. J Periodontol. 2017;88:752–61. 10.1902/jop.2017.160751. 28440740 10.1902/jop.2017.160751

